# Clinical outcomes associated with neoadjuvant therapy for the treatment of resectable non‐small cell lung cancer in real‐world practice

**DOI:** 10.1111/crj.13761

**Published:** 2024-05-01

**Authors:** Xiaojie Huang, Guanchao Pang, Zhirong Mao, Baizhou Li, Zhihua Teng, Yan Yang, Zijian Qiu, Xiuxiu Chen, Pingli Wang

**Affiliations:** ^1^ Department of Respiratory Medicine Second Affiliated Hospital of Zhejiang University, School of Medicine Hangzhou China; ^2^ Department of Respiratory and Critical Care Medicine The Fourth Affiliated Hospital, International Institutes of Medicine, Zhejiang University School of Medicine Yiwu China; ^3^ Department of Pathology Second Affiliated Hospital of Zhejiang University, School of Medicine Hangzhou China; ^4^ Department of Thoracic Surgery Second Affiliated Hospital of Zhejiang University, School of Medicine Hangzhou China; ^5^ Department of Radiotherapy Quzhou People's Hospital Quzhou China

**Keywords:** chemotherapy, disease‐free survival, immunochemotherapy, immunotherapy, neoadjuvant, non‐small cell lung cancer, treatment response

## Abstract

**Background:**

In order to improve survival outcomes in resectable non‐small cell lung cancer (NSCLC), strategies for neoadjuvant therapy need to be revisited. We evaluated and compared the efficacy of different neoadjuvant therapeutic modalities in a real‐world setting.

**Methods:**

A total of 258 patients with clinical stage IIA to IIIB NSCLC was included. All the patients underwent surgical resection after one to four cycles of neoadjuvant treatment consisting of chemotherapy (83), immunotherapy (23), and immunotherapy plus chemotherapy (152).

**Results:**

The radiologic response rate in the combined immunochemotherapy group was 67.8%, higher than that of 48.2% in the chemotherapy group and 4.3% in the immunotherapy group (*p* < 0.001). An improved major pathological response (MPR) was also achieved in the combined therapy group compared with the chemotherapy group and the immunotherapy group (53.9% vs. 10.8% vs. 8.7%, *p* < 0.001). Patients in the combined therapy group had a significant trend toward longer disease‐free survival than those in the chemotherapy alone group (3‐year disease‐free survival [DFS] of 68.79% vs. 50.81%; hazard ratio [HR] for progression or death, 0.477; *p* = 0.003). Multivariate Cox analysis identified radical surgery (HR, 0.328; *p* = 0.033), ypN0–1 stage (HR, 0.591; *p* = 0.038) and MPR result (HR, 0.362; *p* = 0.007) to be independent prognostic factors for DFS.

**Conclusions:**

Neoadjuvant treatment with a combination of immunotherapy plus chemotherapy appears to achieve higher radiological and pathological responses than monotherapy for IIA‐IIIB NSCLC. Log‐rank analysis showed that a better outcome could be expected in patients with the addition of immunotherapy to neoadjuvant chemotherapy if compared with patients with chemotherapy alone in terms of DFS.

## Graphical abstract

The present study evaluated and compared the efficacy of different neoadjuvant therapeutic modalities in patients with operable NSCLC (non‐small cell lung cancer) in a real‐world setting. The results demonstrated that neoadjuvant immunochemotherapy yields better effects in terms of pathologic response and disease‐free survival compared with chemotherapy or immunotherapy alone. This regimen does not increase the risk of adverse events or hinder surgical resection.

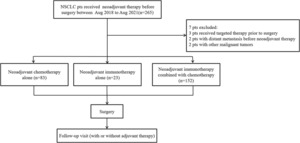

## 1 INTRODUTION

Non‐small cell lung cancer (NSCLC) is the main subtype of lung cancer, which remains the leading cause of cancer death worldwide.^1^ For early and locally advanced stage NSCLC, surgical resection is the preferred treatment to achieve cure.^2^ However, despite a complete surgical excision, most patients will subsequently suffer from disease relapse,^3^ resulting in a 5‐year overall survival (OS) data range from 60% for stage‐IIA patients to 26% for stage‐IIIB patients.^4^

The addition of preoperative neoadjuvant therapy has the potential to reduce tumor size, downstage nodal status,^5^ increase operability,^6^ and eradicate micro‐metastases.^7^ A large meta‐analysis to establish the effect of preoperative chemotherapy for patients with resectable NSCLC showed a significant benefit on survival, with a hazard ratio of 0.87 (95% confidence interval [CI], 0.78–0.96), but the overall survival rate at 5 years was merely increased by 5%.^8^

In recent years, immune‐checkpoint inhibitors (ICIs) targeting programmed death‐1 (PD‐1) and programmed death‐ligand 1 (PD‐L1) have changed the treatment paradigm for patients with advanced‐stage NSCLC. The great success of immunotherapy in advanced diseases has paved the way for the use of ICIs in a neoadjuvant setting, with the theoretical advantage of priming systemic immunity against tumor antigens and eliminating micro‐metastatic tumor deposits.^9^ At present, multiple clinical trials of the neoadjuvant anti‐PD‐1/PD‐L1 blockade, either as monotherapy or in combination with chemotherapy, have demonstrated the efficacy of this approach in patients with resectable (stage I‐IIIA) or potentially resectable (stage IIIB) lung cancer. The LCMC3 trial unraveled a major pathologic response (MPR) rate of 21% after two cycles of atezolizumab in a neoadjuvant regimen, with a disease‐free survival (DFS) rate of 85% at 1 year.^10^ Another single‐arm NADIM study evaluating neoadjuvant nivolumab plus carboplatin and paclitaxel in patients with resectable stage IIIA NSCLC reported an MPR rate of 83%. The 12‐month progression‐free survival (PFS) and OS in NADIM were 95.7% and 97.8% respectively.^11^ CheckMate‐816 was the first phase 3 trial designed to compare preoperative nivolumab plus chemotherapy with chemotherapy alone, with event‐free survival (EFS) and pathological complete response (pCR) as the primary endpoints. Besides showing a significantly higher pCR rate with combination therapy (24.0% vs. 2.2% for chemotherapy alone), significantly longer EFS was also observed with combination therapy (31.6 months vs. 20.8 months for chemotherapy alone).^12^

Growing evidence suggests that the neoadjuvant use of immunotherapy may improve the prognosis of patients with resectable lung cancer, and the treatment with a combination of immunotherapy plus chemotherapy significantly improved MPR compared with immunotherapy alone or chemotherapy alone. However, it is still important to understand both safety and efficacy in real‐world clinical practice. Whether combination therapy compliance is favorable in the preoperative setting in real clinical practice needs to be further verified. Here, we retrospectively reviewed resected NSCLC cases with different neoadjuvant therapy strategies (including neoadjuvant chemotherapy, neoadjuvant immunotherapy and neoadjuvant immunochemotherapy), and aim to explore the differences in clinical efficacy and to investigate the possible prognostic factors. The final results of this real‐world study may have a meaningful effect on the treatment strategies for patients with locally advanced resectable NSCLC.

## 2 METHODS

### 2.1 Patients

NSCLC patients who received neoadjuvant therapy (one cycle at least) before surgical procedure at the Second Affiliated Hospital of Zhejiang University from August 2018 to August 2021 were enrolled in this study. Neoadjuvant therapy included chemotherapy alone, immunotherapy alone and immunotherapy combined with chemotherapy. The neoadjuvant regimen and specific cycles were determined by the physician in charge taking into account the evidence available at that time. The initial patients were treated with neoadjuvant chemotherapy or immuno‐monotherapy, and the most subsequent patients were treated with neoadjuvant immunochemotherapy. Meanwhile, the choice of treatment modalities was also influenced by the patients' concerns about side effects and economic issues. Furthermore, patients not yet eligible for surgery due to poor response to therapy or performance status may receive extended neoadjuvant treatment. Patients who met the following criteria were excluded: (1) pathologically diagnosed as small cell lung cancer; (2) received targeted therapy prior to surgery; (3) distant metastasis existed before neoadjuvant treatment; (4) concurrent with other malignant tumors. Eligible patients received initial evaluation and staging, including enhanced chest computed tomography (CT), brain magnetic resonance imaging (MRI), bone scan, abdominal CT or ultrasound, and/or positron emission tomography‐CT (PET/CT). Clinical staging was performed according to the eighth edition of the TNM classification^4^ with lymph node staging evaluated through endobronchial ultrasound‐guided transbronchial needle aspiration (EBUS‐TBNA) or PET/CT scan. The data on demographic and clinical features were collected from routine clinical records. Follow‐up data were obtained from outpatient reviews or telephone interviews. This retrospective observational study was approved by the institutional review board of the Second Affiliated Hospital of Zhejiang University and informed consent was obtained in accordance with the Declaration of Helsinki.

### 2.2 Treatment and follow‐up

Patients in the chemotherapy group or combined therapy group received conventional platinum‐based doublets chemotherapy every 3 weeks per cycle. The specific chemotherapy regimens were determined according to the pathological type of the tumor, including paclitaxel, pemetrexed, gemcitabine, or docetaxel. Nivolumab (10 cases, 43.5%) or pembrolizumab (13 cases, 56.5%) was given intravenously one or two cycles before the surgery in the immunotherapy alone group. While in the combined therapy group, in addition to the above‐mentioned two kinds of immunotherapy drugs, there also included several domestic PD‐1 inhibitors, such as sintilimab (62 cases, 40.8%), tislelizumab (26 cases, 17.1%), camrelizumab (24 cases, 15.8%), and toripalimab (9 cases, 5.9%).

After proposed cycles of neoadjuvant therapy, the response was evaluated according to the WHO criteria, and the resectability was confirmed by a multi‐disciplinary team of thoracic surgeons, radiologists and oncologists. Surgery was planned approximately 4 weeks after the last neoadjuvant therapy. Complete resection (R0) was achieved in all patients with neoadjuvant immunotherapy alone. Four (4.8%) patients in the chemotherapy group and two (1.3%) patients in the combined therapy group did not undergo R0 resection due to unresectable tumors at the time of exploration or microscopically positive margins.

The pathological response was evaluated by measuring the percentage of residual viable tumor in the primary tumor bed and all resected lymph nodes. MPR was defined as no more than 10% viable residual tumor in the resected specimen, and those with no viable tumor cells in all the pathological slices (including removed lymph nodes) were deemed to be pCR. Moreover, nodal downstaging in this study referred to the difference between the previous clinical N stage and the ypN stage. The administration of adjuvant therapy (radiotherapy, chemotherapy or immunotherapy) after surgery was adopted through the discussion of the attending physicians according to pathological responses and postoperative clinical conditions.

Surveillance after surgery was carried out every 2–3 months for the first 2 years, including physical examinations and chest CT scan (along with abdominal ultrasound and examination of suspected lesions). The disease‐free survival (DFS) was defined as the time from surgery date to recurrence or death for those who died without relapse. Patients who did not have an event were censored at the last follow‐up visit. Overall survival (OS) was calculated from the date of the surgery to the date of death from any cause, with survivors being censored at the time they were last known to be alive.

### 2.3 Statistical analysis

Patients were characterized by median, minimum, and maximum for continuous variables, while frequencies and percentages for categorical ones. Comparisons between the groups were analyzed using the chi‐square test (*χ* ^2^) or Fisher's exact test for categorical variables. The Kruskal–Wallis test was used for grade data and the data that did not obey parameter distribution. DFS was performed by the Kaplan–Meier curves and compared by the log‐rank tests. Univariate and multivariate analyses were performed with the Cox hazards regression model to assess the prognostic factors on patients' recurrence and survival rate. All statistical analyses were performed using SPSS (IBM SPSS Statistics 25) and R V.4.2.1. The *p* values were two‐sided, and data of less than 0.05 indicated a statistically significant difference.

## 3 RESULTS

### 3.1 Patient characteristics

A total of 258 eligible patients with stage IIA to IIIB NSCLC was enrolled in this study, of whom 83 patients received neoadjuvant chemotherapy, 23 patients received neoadjuvant immunotherapy, and 152 patients received neoadjuvant immunochemotherapy. The demographic and baseline patient characteristics are summarized in Table 1. There were no significant differences among the three groups in age and smoking status. However, in terms of gender distribution, the proportion of males in the chemotherapy group or in the combined therapy group is higher than that in the immunotherapy group (*p* = 0.006). The percentage of patients with stage T3–4 disease in the immunotherapy group was 17.4%, which was lower than in the chemotherapy group (39.7%) or in the combined therapy group (41.4%), but the difference was not significant (*p* = 0.188). There was also no significant statistical difference in the clinical N stage among the three groups (*p* = 0.553). The percentages of patients with clinical N2 stage in each group were 52.2%, 56.6%, and 53.9%, respectively. In the chemotherapy group, most patients were diagnosed with stage III disease at presentation (IIIA: 54.2%; IIIB: 18.1%), and the principal pathological type was squamous cell carcinoma (71.1%). A similar distribution was observed in the combined therapy group, 49.3% of patients had stage IIIA disease, and 18.4% of patients had stage IIIB disease, majority of patients (69.1%) were diagnosed as squamous cell carcinoma. In the immunotherapy group, 56.5% of patients had stage IIIA disease, and 4.3% of patients had stage IIIB disease; the main pathological type was adenocarcinoma (52.2%). The difference in histology was statistically significant (*p* = 0.002).

**TABLE 1:** Characteristics of the patients at baseline according to the neoadjuvant treatment.

Characteristics	NCT *n* = 83 (%)	NIT *n* = 23 (%)	NCIT *n* = 152 (%)	*p* value
Age, years				0.051
<60	27 (32.5)	13 (56.5)	47 (30.9)	
≥60	56 (67.5)	10 (43.5)	105 (69.1)	
Sex				**0.006**
Male	74 (89.2)	15 (65.2)	135 (88.8)	
Female	9 (10.8)	8 (34.8)	17 (11.2)	
Smoking status				0.094
Current or ever	59 (71.1)	11 (47.8)	105 (69.1)	
Never	24 (28.9)	12 (52.2)	47 (30.9)	
Clinical tumor stage				0.188
cT1	14 (16.9)	4 (17.4)	15 (9.9)	
cT2	36 (43.4)	15 (65.2)	74 (48.7)	
cT3	19 (22.9)	4 (17.4)	40 (26.3)	
cT4	14 (16.8)	0 (0)	23 (15.1)	
Clinical nodal stage				0.553
N0	12 (14.5)	1 (4.3)	23 (15.1)	
N1	24 (28.9)	10 (43.5)	47 (30.9)	
N2	47 (56.6)	12 (52.2)	82 (53.9)	
Clinical TNM stage				0.612
IIA	2 (2.4)	0 (0)	5 (3.3)	
IIB	21 (25.3)	9 (39.1)	44 (28.9)	
IIIA	45 (54.2)	13 (56.5)	75 (49.3)	
IIIB	15 (18.1)	1 (4.3)	28 (18.4)	
Histology				**0.002**
Squamous	59 (71.1)	9 (39.1)	105 (69.1)	
Adenocarcinoma	14 (16.9)	12 (52.2)	37 (24.3)	
Adenosquamous	3 (3.6)	1 (4.3)	4 (2.6)	
Large cell	7 (8.4)	0 (0)	2 (1.3)	
Others	0 (0)	1 (4.3)	4 (2.6)	
Treatment cycles				**<0.001**
1	2 (2.4)	13 (56.5)	0 (0)	
2	73 (88.0)	10 (43.5)	119 (78.3)	
3	7 (8.4)	0 (0)	32 (21.0)	
4	1 (1.2)	0 (0)	1 (0.7)	
Interval time, days^a^				0.225
≤42 days	75 (90.4)	23 (100)	143 (94.1)	
>42 days	8 (9.6)	0 (0)	9 (5.9)	
Surgery approach				0.430
VATS	67 (80.7)	21 (91.3)	131 (86.2)	
Conversion	9 (10.9)	2 (8.7)	16 (10.5)	
Thoracotomy	7 (8.4)	0 (0)	5 (3.3)	
Extent of resection				0.088
Lobectomy	54 (65.1)	22 (95.7)	99 (65.1)	
Bilobectomy	15 (18.1)	1 (4.3)	34 (22.4)	
Pneumonectomy	2 (2.4)	0 (0)	5 (3.3)	
Sleeve	12 (14.4)	0 (0)	14 (9.2)	
Surgical radicality				0.255
Radical	79 (95.2)	23 (100)	150 (98.7)	
Palliative	4 (4.8)	0 (0)	2 (1.3)	

*Note*: Data are expressed as *n* (%). Factors with *p* values less than 0.05 are presented in bold.

Abbreviations: NCT, neoadjuvant chemotherapy; NCIT, neoadjuvant chemo‐immunotherapy; NIT, neoadjuvant immunotherapy; VATS, video‐assisted thoracic surgery.

^a^ Interval between final neoadjuvant therapy and surgery.

### 3.2 Efficacy and surgery

**FIGURE 1:**
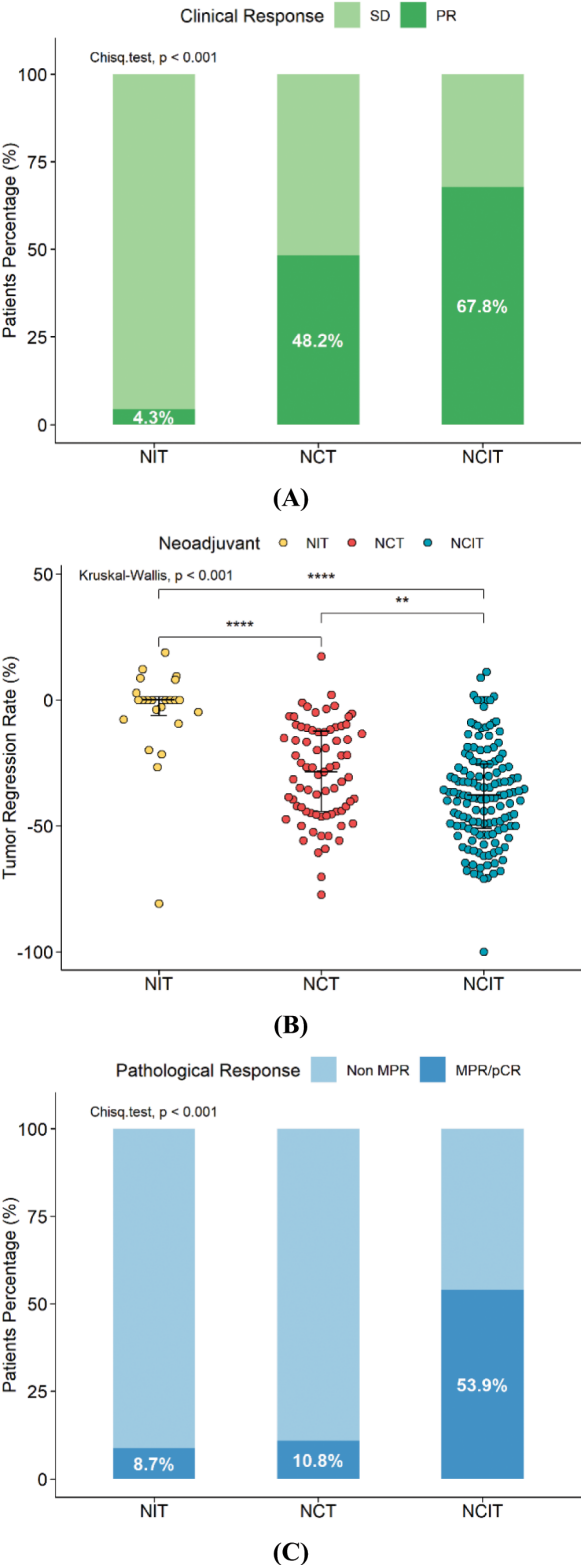
Clinical efficacy after neoadjuvant therapy. (A) Radiological assessment of response to neoadjuvant therapy. (B) Tumor regression rate after different neoadjuvant therapy type. (C) Pathological assessment of response to neoadjuvant therapy.

After one to four cycles of neoadjuvant therapy, 67.8% of patients achieved PR (partial response) in the combined therapy group, which was more than that in the chemotherapy group (48.2%) and in the immunotherapy group (4.3%) (Figure 1A, *p* < 0.001). No patients were observed with PD (progressive disease) in all three groups. In terms of tumor regression rate, patients who received neoadjuvant immunochemotherapy had a higher tumor regression rate as compared with those who received neoadjuvant monotherapy (Figure 1B, *p* < 0.001).

Adverse events (AEs) during neoadjuvant therapy were graded according to the Common Terminology Criteria for Adverse Events, version 5.0 (CTCAE5.0) (Table 2). There were fewer AEs observed in the immunotherapy alone group. While in another two groups, the most common AEs included anemia, decreased white blood cells and neutrophils count, decreased platelet count, and increased alanine aminotransferase. The recorded AEs were mainly grade 1 or 2 and manageable. Eight patients (9.6%) in the chemotherapy group and nine patients (5.9%) in the combined therapy group underwent surgery beyond 42 days after the final neoadjuvant therapy (Table 1). The delay was mainly due to hesitation to undergo surgery, while two patients from the combined therapy group received delayed surgery attributed to neutropenia and pulmonary infection. Most patients received pulmonary resection via minimally invasive surgery (video‐assisted thoracic surgery [VATS]); the percentages were 80.7% in the chemotherapy group, 91.3% in the immunotherapy group, and 86.2% in the combined therapy group respectively, while 10.9%, 8.7%, and 10.5% of the patients in each group received conversion thoracotomy because of the development of dense fibrosis and adhesions. In addition, seven patients in the chemotherapy group and five patients in the immunochemotherapy group received traditional open thoracotomy. Overall, there were no significant differences among the three groups in the surgery approach (*p* = 0.430) and the extent of resection (*p* = 0.088). Only one patient in the combined therapy group died 7 days after surgery due to bronchial anastomotic leakage and hemoptysis. No other 90‐day postoperative mortality occurred in the three groups.

**TABLE 2:** Treatment‐related adverse events.

Adverse events: *n* (%)	NCT (*n* = 83)			NIT (*n* = 23)			NCIT (*n* = 152)		
	Grade 1–2	Grade 3	Grade 4	Grade 1–2	Grade 3	Grade 4	Grade 1–2	Grade 3	Grade 4
White blood cells decreased	32 (38.6)	4 (4.8)	1 (1.2)	0	0	0	49 (32.2)	3 (2)	0
Neutrophil count decreased	25 (30.1)	6 (7.2)	3 (3.6)	0	0	0	41 (27)	5 (3.3)	2 (1.3)
Anemia	58 (69.9)	1 (1.2)	0	3 (13)	0	0	109 (71.7)	3 (2)	0
Platelet count decreased	10 (12)	0	0	0	0	0	22 (14.5)	2 (1.3)	1 (0.7)
Alanine aminotransferase increased	23 (27.7)	0	0	4 (17.4)	0	0	51 (33.6)	2 (1.3)	0
Creatinine increased	3 (3.6)	0	0	0	0	0	7 (4.6)	0	0
Diarrhea	1 (1.2)	0	0	0	0	0	3 (2)	0	0
Rash	2 (2.4)	0	0	1 (4.3)	0	0	12 (7.9)	0	0
Mucositis oral	1 (1.2)	0	0	0	0	0	7 (4.6)	0	0
Pneumonitis	2 (2.4)	0	0	0	0	0	5 (3.3)	0	0

*Note*: Data are expressed as *n* (%).

Abbreviations: NCT, neoadjuvant chemotherapy; NCIT, neoadjuvant chemo‐immunotherapy; NIT, neoadjuvant immunotherapy.

Regarding the evaluation of the pathological response, the MPR rate in the combined therapy group was 53.9%, including 38.2% with pCR, which was significantly higher than that in the chemotherapy group (MPR rate of 10.8%, *p* < 0.001; pCR rate of 7.2%) and immunotherapy group (MPR rate of 8.7%, *p* < 0.001; pCR rate of 4.3%) (Figure 1C). Moreover, the descending rate of the lymph node in the combined therapy group was significantly higher than that in the other two groups (Table 3, *p* = 0.002), and ypN0 stage was most often seen in the combined therapy group (*p* = 0.001). Pathological examination revealed persistent N2 after neoadjuvant therapy in 18 patients (11.8%) in the combined therapy group, 7 patients (30.4%) in the immunotherapy group, and 26 patients (31.3%) in the chemotherapy group. Postoperative adjuvant therapy was administered in 71 cases (85.5%) in the chemotherapy group, 13 cases (56.5%) in the immunotherapy group, and 138 cases (90.8%) in the combined therapy group (*p* < 0.001).

**TABLE 3:** Outcomes according to the neoadjuvant treatment.

	NCT *n* = 83 (%)	NIT *n* = 23 (%)	NCIT *n* = 152 (%)	*p* value
Clinical response				**<0.001**
Partial response	40 (48.2)	1 (4.3)	103 (67.8)	
Stable response	43 (51.8)	22 (95.7)	49 (32.2)	
ypN stage				**0.001**
N0	41 (49.4)	11 (47.8)	111 (73.0)	
N1	16 (19.3)	5 (21.7)	23 (15.1)	
N2	26 (31.3)	7 (30.4)	18 (11.8)	
Nodal downstaging				**0.002**
Yes	34 (41.0)	13 (56.5)	98 (64.5)	
No	49 (59.0)	10 (43.5)	54 (35.5)	
Tumor downstaging				**<0.001**
Yes	38 (45.8)	4 (17.4)	102 (67.1)	
No	45 (54.2)	19 (82.6)	50 (32.9)	
Pathological response				**<0.001**
MPR	9 (10.8)	2 (8.7)	82 (53.9)	
pCR	6 (7.2)	1 (4.3)	58 (38.2)	
Non‐MPR	74 (89.2)	21 (91.3)	70 (46.1)	
Adjuvant systemic therapy				**<0.001**
Yes	71 (85.5)	13 (56.5)	138 (90.8)	
No	12 (14.5)	10 (43.5)	14 (9.2)	
Recurrence				**<0.001**
Yes	37 (44.6)	7 (30.4)	24 (15.8)	
No	46 (55.4)	16 (69.6)	128 (84.2)	
First failure site				0.456
Local	16 (19.3)	2 (8.7)	13 (8.6)	
Distant	21 (25.3)	5 (21.7)	11 (7.2)	
Brain	7 (8.4)	2 (8.7)	4 (2.6)	
Adrenal gland	2 (2.4)	0 (0)	1 (0.7)	
Liver	2 (2.4)	0 (0)	3 (2.0)	
Bone	4 (4.8)	0 (0)	0 (0)	
Contralateral lung	4 (4.8)	2 (8.7)	2 (1.3)	
SC lymph nodes	2 (2.4)	1 (4.3)	1 (0.7)	

*Note*: Data are expressed as *n* (%). Factors with *p* values less than 0.05 are presented in bold.

Abbreviations: MPR, major pathological response; NCT, neoadjuvant chemotherapy; NCIT, neoadjuvant chemo‐immunotherapy; NIT, neoadjuvant immunotherapy; non‐MPR, major pathological response not reached; pCR, pathological complete response; SC, supraclavicular.

### 3.3 DFS outcomes and prognostic factors (subgroup analysis)

**FIGURE 2:**
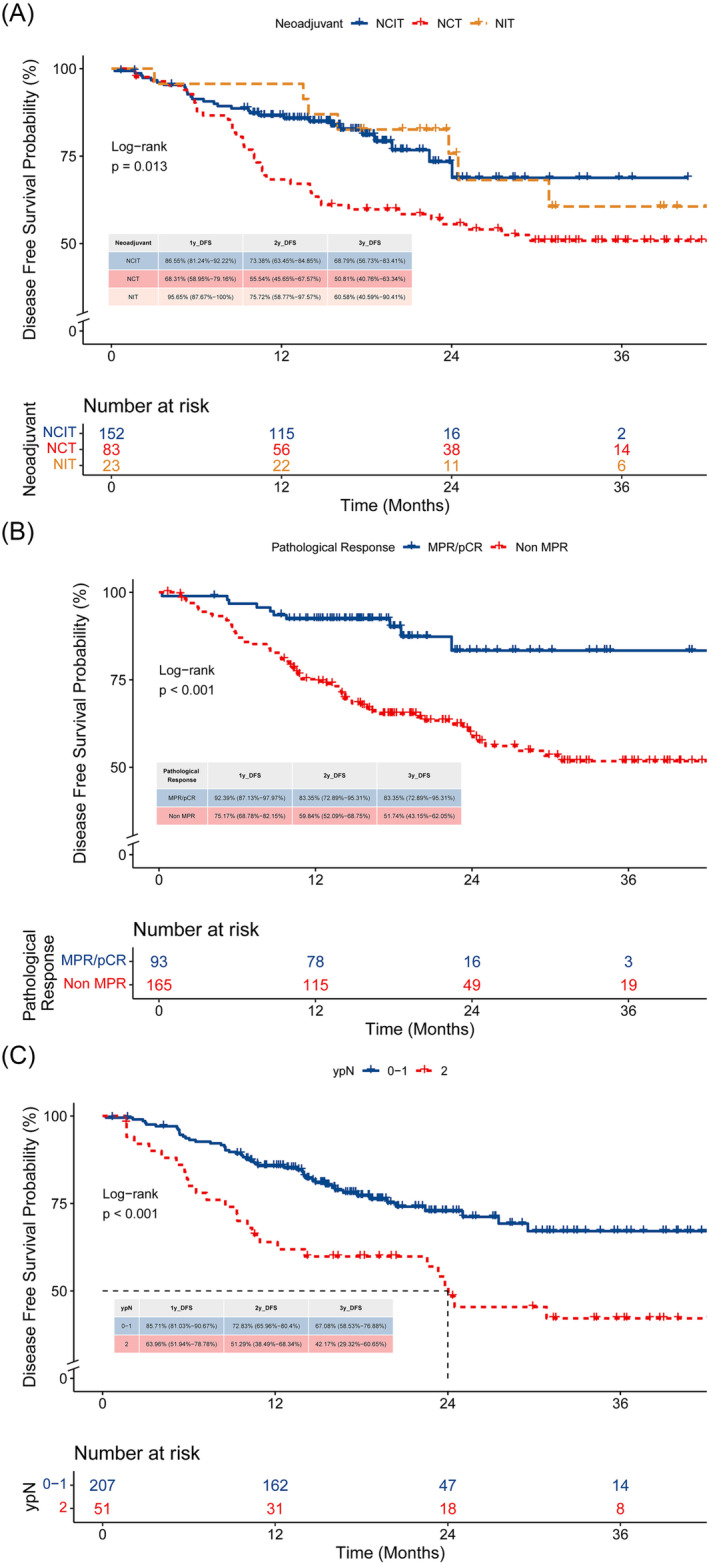
(A) Kaplan–Meier curves of DFS according to different neoadjuvant treatment type. (B) Kaplan–Meier curves of DFS for different pathological response. (C) Kaplan–Meier curves of DFS for lymph node staging status after neoadjuvant therapy.

**FIGURE 3:**
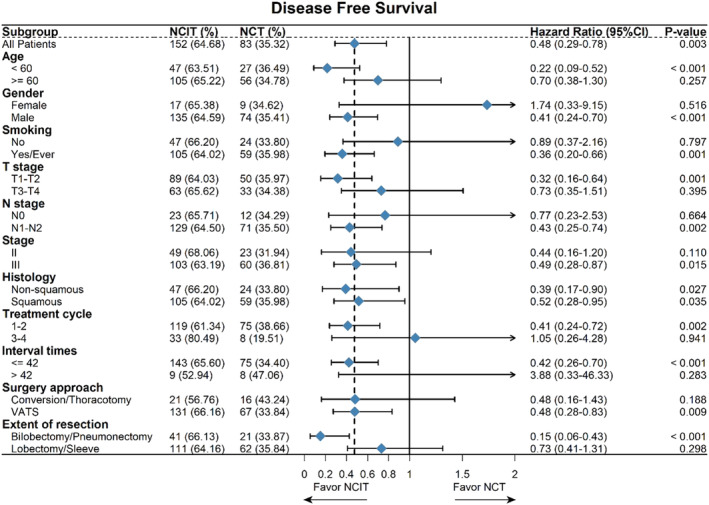
Hazard ratios for disease‐free survival in subgroups of patients for the neoadjuvant immunochemotherapy group compared with the chemotherapy group.

After a maximum of 45.1 months of follow‐up (median of 19.2 months), there were 39 (47.0%) events in 83 patients from the chemotherapy group (37 relapses, 2 deaths from causes other than lung cancer), 7 (30.4%) events in 23 patients from the immunotherapy group (7 relapses), and 29 (19.1%) events in 152 patients from the combined therapy group (24 relapses, 5 deaths from causes other than lung cancer). The first recurrent sites included local recurrence and distant metastasis (of which the brain metastasis was more often), and there was no significant difference among the three groups (*p* = 0.456). In the patients who achieved pCR after neoadjuvant treatment, recurrences were observed in two patients (3.1%) during the follow‐up period. We performed a Kaplan–Meier survival analysis on the three groups (Figure 2A). Although the median DFS time had not been reached at the time of data analysis, statistical differences still existed among the three groups (log‐rank *p* = 0.013). The 1‐year DFS rates were 68.31% in the chemotherapy group, 95.65% in the immunotherapy group, and 86.55% in the combined therapy group; the 2‐year DFS rates were 55.54%, 75.52%, and 73.38%, respectively; the 3‐year DFS rates were 50.81%, 60.58%, and 68.79%, respectively. Additionally, compared with the chemotherapy group, the HR for progression or death for the immunotherapy group was 0.543 (95% CI: 0.243–1.216; *p* = 0.138), and the HR for the combined therapy group was 0.477 (95% CI: 0.292–0.780; *p* = 0.003) by Cox regression. Further analysis of DFS in the immunochemotherapy group and chemotherapy group revealed a consistent benefit from immunochemotherapy for most subgroups listed in Figure 3.

For the whole population, univariate Cox analysis demonstrated that the radiographic tumor response, pathological response, radical surgery, and ypN stage were also significant predictors for DFS (Table 4). When these factors were included in multivariate analysis, it showed that radical surgery (HR = 0.328, 95% CI: 0.118–0.913, *p* = 0.033), ypN0–1 stage (HR = 0.591, 95% CI: 0.360–0.972, *p* = 0.038), and MPR result (HR = 0.362, 95% CI: 0.174–0.753, *p* = 0.007) remained independent factors associated with better prognosis. Kaplan–Meier analysis of DFS according to the pathological response was depicted in Figure 2B, showing that the MPR group had a greater DFS benefit compared with the non‐MPR group (the 3‐year DFS rate: 83.35% vs. 51.74%, log‐rank *p* < 0.001). In terms of lymph node involvements after neoadjuvant therapy, patients with persistent N2 status (ypN2) had a worse prognosis compared with those with ypN0–1 (the median DFS time: 24 months vs. not reached, log‐rank *p* < 0.001), as reported in Figure 2C.

**TABLE 4:** Univariate and multivariate analysis of DFS for the overall populations.

Characteristics	Univariate		Multivariate	
	HR (95% CI)	*p* value	HR (95% CI)	*p* value
Age, years				
<60	Reference	‐	‐	‐
≥60	0.923 (0.578–1.474)	0.737	‐	‐
Sex				
Female	Reference	‐	‐	‐
Male	1.320 (0.657–2.652)	0.435	‐	‐
Smoking status				
Current or ever	Reference	‐	‐	‐
Never	0.994 (0.615–1.607)	0.981	‐	‐
Clinical tumor stage				
cT1–2	Reference	‐	‐	‐
cT3–4	1.395 (0.881–2.208)	0.156	‐	‐
Clinical nodal stage				
N0	Reference	‐	‐	‐
N1–2	0.869 (0.458–1.649)	0.667	‐	‐
Clinical TNM stage				
IIA‐IIB	Reference	‐	‐	‐
IIIA‐IIIB	1.706 (0.994–2.931)	0.053	‐	‐
Histology				
Squamous	Reference	‐	‐	‐
Non‐squamous	1.314 (0.825–2.091)	0.250	‐	‐
Treatment cycles				
1–2	Reference	‐	‐	‐
3–4	1.091 (0.573–2.075)	0.791	‐	‐
Interval time, days				
≤42 days	Reference	‐	‐	‐
>42 days	0.461 (0.145–1.464)	0.189	‐	‐
Clinical response^a^				
Stable response	Reference	‐	Reference	‐
Partial response	0.573 (0.361–0.908)	**0.018**	0.723 (0.441–1.187)	0.200
Neoadjuvant therapy^a^				
Chemotherapy	Reference	‐	Reference	‐
Immunotherapy	0.551 (0.246–1.233)	**0.147**	0.473 (0.206–1.084)	0.077
Immunochemotherapy	0.495 (0.302–0.810)	**0.005**	0.827 (0.488–1.402)	0.481
Surgery approach				
VATS	Reference	‐	‐	‐
Conversion/thoracotomy	1.504 (0.865–2.615)	0.148	‐	‐
Extent of resection				
Lobectomy/sleeve	Reference	‐	‐	‐
Bilobectomy/pneumonectomy	1.080 (0.629–1.857)	0.780	‐	‐
Surgical radicality^a^				
Palliative	Reference	‐	Reference	‐
Radical	0.237 (0.086–0.650)	**0.005**	0.328 (0.118–0.913)	**0.033**
ypN stage^a^				
N2	Reference	‐	Reference	‐
N0–1	0.449 (0.278–0.724)	**0.001**	0.591 (0.360–0.972)	**0.038**
Pathological response^a^				
Non‐MPR	Reference	‐	Reference	‐
MPR	0.271 (0.139–0.529)	**<0.001**	0.362 (0.174–0.753)	**0.007**

Abbreviations: CI, confidence interval; DFS, disease‐free survival; HR, hazard ratio; MPR, major pathological response; non‐MPR, major pathological response not reached; VATS, video‐assisted thoracic surgery.

^a^ Factors included into multivariate analysis.

*Note*: Factors with meaningful *p* values that were included into multivariate analysis are presented in bold.

## 4 DISCUSSION

Surgery alone for patients with locally advanced stage NSCLC is not sufficient to improve their prognosis due to the high rate of local and distant recurrence, while neoadjuvant therapies followed by surgery may provide better clinical outcomes by eradicating micro‐metastases in the early phases. Moreover, encouraged by the success of the immunotherapy approach in advanced stage NSCLC, the incorporation of immunotherapy in the neoadjuvant setting has been an important development in recent years. However, although multiple studies have preliminarily confirmed the efficacy of neoadjuvant immunotherapy, there is still a lower effective rate and survival rate.^13^, ^14^, ^15^ Based on the hypothesis that “cytotoxic chemotherapy can increase the therapeutic effects of immunotherapy by releasing tumor antigens from cancer cell death,”^16^ a growing number of neoadjuvant trials using immunotherapy combined with chemotherapy are carried out.^17^ In this study, we retrospectively analyzed real‐world cases with neoadjuvant therapy and revealed encouraging results consistent with previous studies. The neoadjuvant immunotherapy plus chemotherapy group showed a significantly higher pathological response rate when compared with the cytotoxic chemotherapy group or ICI monotherapy group, with an MPR rate of 53.9% and a pCR rate of 38.2%, respectively. It has been widely accepted that the percentage of viable tumor cells in the resected specimen was recognized as a surrogate endpoint for overall survival, and MPR was associated with improved survival.^18^ Reviewing the clinical trials published in the literature, the rate of MPR for patients who received neoadjuvant chemotherapy was reported to be 16%.^19^ In the monotherapy‐ICI cohort, the MPR rates ranged from 21% to 45%,^10^, ^13^ while none of the patients showed an MPR after one dose of neoadjuvant atezolizumab in the PRINCEPS trial.^20^ Neoadjuvant immunochemotherapy combinations were explored in various clinical trials, with MPR rates exceeding 80% and pCR rates exceeding 60%.^11^, ^21^ A large phase 3 trial of Checkmate 816 showed the improved MPR and pCR rates with the addition of nivolumab to neoadjuvant chemotherapy (36.9% vs. 8.9% and 24.0% vs. 2.2% for chemotherapy alone, respectively).^12^ Consistent with the above results, our data from a real‐world setting further confirmed that neoadjuvant immunochemotherapy yielded promising efficacy, with a 43.1% and a 45.2% increased MPR rate compared with chemotherapy alone and immunotherapy alone, which may be explained by the synergistic effect of chemotherapy and ICIs. Additionally, patients observed in this retrospective study had a wider range of clinical stages, ranging from stage IIA to stage IIIB, and there are also several PD‐1 inhibitor alternatives besides nivolumab. To the best of our knowledge, we are unaware of mature clinical trials of immunotherapy drugs other than nivolumab for the neoadjuvant therapy of NSCLC. Our results uncovered that other PD‐1 inhibitors may also have promising efficacy similar to that of nivolumab, highlighting the potential of using other ICIs in routine clinical practice. Moreover, we observed that the pathological response was inconsistent with the radiographic response. Thirteen of 58 (22.4%) patients with pathological complete responses in the immunochemotherapy group were classified as non‐responders by posttreatment CT evaluation. This phenomenon has been previously documented^22^ and is likely due to the presence of tumor‐infiltrating lymphocytes and fibrosis in the tumor bed. Thus, effective identification of this discrepancy between imageology and pathology is crucial for accurate assessment of the degree of tumor regression and may avoid subsequent surgery.

In general, neoadjuvant cancer therapies may offer the potential clinical advantage of increasing resectability due to tumor shrinkage. In our study, higher tumor downstaging rates (67.1%) were observed in the combined therapy group compared to 45.8% and 17.4% in another two groups, indicating that immunochemotherapy can dramatically shrink the primary tumor and make radical minimally invasive surgery feasible. However, it should also be noted that mediastinal and hilar dense adhesions or fibrosis may develop as a result of response to neoadjuvant therapy, and it may increase the surgical difficulty and result in more conversions to thoracotomy.^23^ In the current study, the rates of conversion to thoracotomy appeared to be similar among the three groups (10.9% in the chemotherapy group, 8.7% in the immunotherapy group, and 10.5% in the combined therapy group). Of note, all of these cases underwent complete resection and did not develop more postoperative complications.

During the process of neoadjuvant therapy, the treatment‐related adverse events (TRAE) should be paid to attention, which may result in delayed or inoperable surgery and increase perioperative morbidity and mortality.^24^ In previous clinical trials of neoadjuvant ICI monotherapy, the proportion of failure to undergo surgery ranged from 0 to 12%, and the incidence of TRAE of ≥G3 was 4.5% to 14%.^10^, ^14^, ^25^ The Checkmate 816 study, which used nivolumab plus chemotherapy, reported 16% of patients failed to undergo surgery and 19% of patients encountered grade 3–4 TRAE. The proportions were similar to those with chemotherapy alone (21% and 21%, respectively). Moreover, there were 6 (4%) and 9 (7%) patients, respectively, who received surgery delayed beyond 42 days due to AEs in the ICI plus chemotherapy and chemotherapy groups.^12^ Thus, there exists an urgent need to identify the benefit–risk profile in large cohort real‐world settings. Here in our study, the incidence of TRAEs was low, and most were grade 1 or 2. In addition, we reported eight patients (9.6%) with delayed surgery in the chemotherapy group and nine (5.9%) in the combined therapy group. The delay was mainly due to hesitation to undergo surgery, while two of them from the combined therapy group had the reason of neutropenia and pulmonary infection. The ratio of the patients who failed to receive surgery after neoadjuvant therapy was unavailable because they were not included in our study. Regarding the perioperative morbidity and mortality, although one patient with sleeve lobectomy in the combined therapy group experienced serious postoperative complications, the overall treatment‐related toxicity was still acceptable and no new safety signals were identified.

Based on our results, although the follow‐up time was limited and the DFS data was immature, we still observed a statistical difference among the three groups (log‐rank *p* = 0.013). The combined therapy group had the highest 3‐year DFS rate (68.79%), which was 8.21% and 17.98% higher than the immunotherapy group and chemotherapy group. It also needs to be concerned that the neoadjuvant immunotherapy alone group had the highest 1‐year and 2‐year DFS rates, which was probably due to the relatively smaller proportions of patients with clinical IIIB stage included in this group, and such patients were more likely to suffer from recurrence and metastasis. In addition, there were only 23 patients included in the immunotherapy group, which was far less than the number of patients in the other two groups. Overall, immunotherapy plus chemotherapy improved disease‐free survival versus chemotherapy alone with an HR of 0.477, and a consistent DFS benefit was observed for combined therapy in most of the subgroups, including different histology and clinical TNM stage. In multivariate Cox analysis of DFS outcome, we revealed that radical surgery, ypN0–1 stage and MPR result were independent factors associated with better prognosis. Those patients who achieved obvious pathological remission and ypN0–1 status could obtain more survival compared to those with non‐MPR and ypN2. Although the type of neoadjuvant therapy was not an independent factor when concluded in multivariate Cox analysis, immunochemotherapy could significantly improve the pathological response rate and the descending rate of lymph nodes, indicating that the addition of immunotherapy into the neoadjuvant chemotherapy could indirectly affect the prognosis of patients in our study.

Notably, patients with pathological complete responses were still at risk of recurrence. In current study, two of the 65 patients who achieved pCR experienced disease relapse until the last follow‐up. In another retrospective study,^26^ Filippo Lococo et al reported an overall recurrent rate of 51.6% during a median follow‐up time of 56.2 months on a large cohort of patients with pCR after induction therapy and surgery. They also demonstrated that adjuvant therapy should be considered in this subset of patients to achieve long‐term survival. Thus, regular follow‐up should be performed in patients even with complete pathological remission.

Our study still has some limitations that should be noted. First, selection biases were inevitable and statistical power was limited due to the retrospective nature of this study and the varied sample size among the three groups. Second, although the immunotherapy drugs administered in this study were all PD‐1 inhibitors, they might differ in efficacy and safety, and subgroup analysis needs to be performed. Third, the follow‐up time was relatively too short to generate mature DFS and OS data, and the OS as a meaningful endpoint was not analyzed. Therefore, large‐scale randomized clinical trials should be performed to clarify the benefit of neoadjuvant combination therapy in resectable NSCLC, and hopefully, the addition of immunotherapy will eventually change the overall treatment mode of this subset of patients in the future.

In conclusion, this real‐world study confirmed that neoadjuvant immunochemotherapy yields better effects in terms of pCR, MPR and DFS in patients with operable locally advanced NSCLC compared with chemotherapy or immunotherapy alone. This regimen does not increase perioperative morbidity and mortality, which further supports the use of immunotherapy plus chemotherapy in neoadjuvant treatment. Furthermore, our study shows the necessity of long‐term follow‐up in patients even with pCR.

## AUTHOR CONTRIBUTIONS

Pingli Wang designed the study. Zhirong Mao and Zijian Qiu performed the patient selection process and acquired the data. Xiaojie Huang, Guanchao Pang, and Xiuxiu Chen designed the statistical analysis plan and performed the analyses. Xiaojie Huang and Guanchao Pang interpreted the results and prepared the manuscript. Baizhou Li, Zhihua Teng, and Yan Yang provided executive support and supervised the development of the work. All authors reviewed the manuscript critically and approved the content.

## CONFLICT OF INTEREST STATEMENT

The authors declare that they have no conflicts of interests.

## ETHICS STATEMENT

This study was approved by the Institutional Review Board of the Second Affiliated Hospital of Zhejiang University and informed consent was obtained in accordance with the Declaration of Helsinki.

## Data Availability

The datasets used or analyzed during this study are available on reasonable request from the corresponding author.
